# Fluorescence *in situ* hybridization status of voided urine predicts invasive and high-grade upper tract urothelial carcinoma

**DOI:** 10.18632/oncotarget.15344

**Published:** 2017-02-15

**Authors:** Xiaohong Su, Han Hao, Xuesong Li, Zhisong He, Kan Gong, Cuijian Zhang, Lin Cai, Qian Zhang, Lin Yao, Yi Ding, Yanqing Gong, Dong Fang, Zheng Zhang, Liqun Zhou

**Affiliations:** ^1^ Department of Urology, Peking University First Hospital, Xicheng, Beijing, China; ^2^ Institute of Urology, Peking University, National Urological Cancer Center, Beijing, China

**Keywords:** fluorescence *in situ* hybridization (FISH), upper tract urothelial carcinoma (UTUC), high grade, radical nephroureterectomy (RNU), conservative management

## Abstract

Here, we assessed the diagnostic accuracy of fluorescence *in situ* hybridization (FISH) for detecting aggressive upper tract urothelial carcinoma (UTUC). A total of 210 UTUC patients from a single hospital in China were enrolled in this retrospective study between 2012 and 2016. Patients were classified as FISH-positive or -negative based on FISH analysis of voided urine, and the clinicpathological characteristics of these two patient groups were compared. Patients with positive FISH results had more advanced tumor stages and higher tumor grades than those with negative results. The overall sensitivity of FISH for detecting advanced UTUC was 62.4% (131/210). The sensitivity and positive predictive values of positive FISH results were 76.5% and 59.5% for high-grade UTUC and 71.7% and 58.0% for muscle-invasive UTUC. These data suggest that voided urine FISH results accurately predict advanced UTUC and might help distinguish patients with aggressive disease from those who might benefit from conservative management.

## INTRODUCTION

Upper tract urothelial carcinomas (UTUCs) are relatively rare compared to bladder cancer and account for approximately 5% to 10% of urothelial carcinomas [1]. Radical nephroureterectomy (RNU) with an ipsilateral bladder cuff excision, which is the gold-standard treatment for UTUC [2], can result in varying degrees of renal insufficiency. Therefore, conservative management of small, low-grade and non-invasive UTUC may help preserve renal function in select groups of patients [2], and accurate pre-oprative risk stratification of UTUC patients is crucial. However, accurately determining the stage and grade of UTUC via urine cytology or preoperative imaging is difficult. In addition, neither ureteroscopy nor urine cytology is sufficient for accurately predicting muscle invasion or the risk of high-grade disease in UTUC patients [3, 4]. Thus, novel, more reliable methods are needed to identify low-risk patients before surgery.

Fluorescence *in situ* hybridization (FISH) analysis of voided urine has become a popular aid for initial diagnosis and subsequent monitoring for recurrence in bladder cancer patients, and previous studies have confirmed that it is highly sensitive and specific [5, 6]. Here, we investigated the ability of FISH to predict high grade and muscle-invasive tumors, and thus to distinguish tumors for which conservative approaches to treatment would be appropriate from those that should be treated with RNU.

## RESULTS

The clinical and pathological characteristics of the patients are listed in Table 1 and are stratified according to the FISH results. There were 102 male and 108 female patients with a median age of 70.5 years (inter-quartile range [IQR]: 63–75 years). The diagnosis of urothelial carcinoma was pathologically confirmed in all patients; 104 had non-muscle-invasive diseases (≤ pT1), and 106 had muscle-invasive diseases (≥ pT2), while 108 had low grade and 102 had high grade diseases. Conservative surgeries were performed in 21 patients; 7 of the patients had non-muscle-invasive (≤ pT1) and 14 had muscle-invasive (≥ pT2) UTUC, and 12 had low grade and 9 had high grade diseases.

**Table 1:** Clinical and pathological characteristics of UTUC patients according to FISH status

		FISH		Chi-square or Z	*p* value
		Positive	Negative		
Median age		68.81 ± 10.35	67.10 ± 9.52	−1.460	0.144
Gender	Male	70	32	3.298	0.069
	Female	61	47		
Hydronephrosis	Present	20	15	0.491	0.483
	Absent	111	64		
Tumor location	Pelvis	63	29	3.059	0.217
	Ureter	60	46		
	Both	8	4		
Tumor stage	A	6	11	13.709	0.008
	1	49	38		
	2	32	18		
	3	43	11		
	4	1	1		
Tumor grade	High	78	24	16.778	< 0.001
	Low	53	55		
Lymph node status	cN0 or pN0	123	77	1.389	0.239
	N +	8	2		
LVI	Presence	20	9	0.622	0.430
	Absence	111	70		
Tumor size	≤ 3 cm	72	45	0.080	0.777
	> 3 cm	59	34		
Multifocal	Yes	24	10	1.164	0.281
	No	107	69		

FISH = fluorescence *in situ* hybridization; LVI = lymphovascular invasion.

FISH analysis of voided urine from 131 patients returned “positive” results, indicating the presence of chromosomal abnormalities, while FISH results for the remaining 79 patients were negative. No significant differences in age, gender, hydronephrosis, tumor location, lymph node status, lymphovascular invasion (LVI), tumor size, or tumor focality were observed between the positive and negative FISH groups (all *p* > 0.05). However, patients with positive FISH results had more advanced tumor stages (*p* = 0.008) and higher tumor grades than negative patients (*p* < 0.001). In binary logistic regression, after adjusting for clinical and pathological factors, only preoperative positive FISH status was an independent risk factor for high-grade or muscle-invasive UTUC (Table 2).

**Table 2:** Preoperative factors that predict high-risk UTUC in univariate and multivariate analyses

Preoperative factors		Tumor Stage		Univariate	Multivariate		Tumor Grade		Univariate	Multivariate	
		Ta-1	≥ pT2	*p*	HR (95% CI)	*p*	Low grade	High grade	*p*	HR (95% CI)	*p*
Patients		104	106				108	102			
Gender	Male	45	57	0.128	1.233 (0.695−2.189)	0.474	48	54	0.218	1.244 (0.705−2.194)	0.451
	Female	59	49				60	48			
Age	≤ 70	54	51	0.581	1.166 (0.662−2.055)	0.594	57	48	0.407	1.234 (0.696−2.189)	0.472
	> 70	50	55				51	54			
FISH	Positive	55	76	0.005	2.266 (1.272−4.036)	0.005	53	78	< 0.001	3.373 (1.864−6.102)	< 0.001
	Negative	49	30				55	24			
Hydronephrosis	Present	14	21	0.217	1.674 (0.782−3.586)	0.185	19	16	0.711	0.936 (0.425−2.058)	0.869
	Absent	90	85				89	86			
Tumor location	Pelvis	50	42	0.446	0.770 (0.471−1.261)	0.300	45	47	0.563	1.177 (0.733−1.891)	0.500
	Ureter	49	57				58	48			
	Both	5	7				5	7			
Tumor size	≤ 3 cm	65	52	0.05	1.741 (0.993−3.050)	0.053	62	55	0.611	1.083 (0.601−1.953)	0.791
	> 3 cm	39	54				46	47			
Multifocal	Yes	16	18	0.754	0.985 (0.443−2.190)	0.971	16	18	0.578	1.014 (0.454−2.262)	0.973
	No	88	88				92	84			

HR = harzard ratio; CI = confidence interval; FISH = fluorescence *in situ* hybridization.

FISH afforded an overall sensitivity of 62.4% (131/210) in all of the UTUC patients; the FISH status of patients after stratification by both pathological stage and grade is shown in Table 3. Positive FISH results had a sensitivity and PPV of 76.5%% and 59.5% for high-grade UTUC and of 71.7% and 58.0% for muscle-invasive UTUC, respectively. Among the 79 patients with negative preoperative FISH results, 30.4% had high-grade and 36.7% had muscle-invasive UTUC upon final pathology.

**Table 3:** Sensitivity and positive predictive value of positive FISH status for predicting high-grade and muscle-invasive UTUC

	Voided urine FISH (*n* = 210)
High-grade cancer	
Sensitivity	76.47 (67.04–84.31)
Positive predictive value	59.54 (50.62–68.02)
Muscle-invasive cancer (≥ pT2)	
Sensitivity	71.70 (62.12–80.02)
Positive predictive value	58.02 (49.08–66.58)

## DISCUSSION

Conservative management of low-risk UTUCs (which are characterized by unifocal tumors, tumor sizes < 1 cm, low grade tumors, and no evidence of infiltrative lesions on CT urography) allows the renal unit to be preserved and spares the patient from morbidity associated with radical surgery [2]. The size of a tumor and whether it is unifocal or multifocal can be easily determined via preoperative imaging. However, it is difficult to accurately determine the stage and grade of UTUC using currently available methods. For example, Messer *et al*. demonstrated in a multi-institutional study of 326 patients that urine cytology alone was also unable to accurately predict muscle-invasive or high-grade disease in UTUC patients; positive urinary cytology had a sensitivity and PPV of only 56% and 54% for high-grade UTUC, and 62% and 44% for muscle-invasive UTUC, although restricting analysis to patients with selective ureteral cytology improved the diagnostic accuracy [4]. Although ureteroscopy is more sensitive and specific in diagnosing UTUC than urine cytology and radiography [7], it may not be accurate for predicting tumor grade and stage. In an early study, Keeley *et al*. demonstrated that ureteroscopy accurately predicted true tumor grade and stage as indicated by open surgical specimens of upper tract transitional cell carcinoma [8]. However, Straub *et al*. [3] found that the accuracy of preoperative ureteroscopy in predicting the correct tumor grade in UTUC patients was only 58%; this accuracy only increased to 68% when urinary cytology was considered as well. Furthermore, ureteroscopy is an invasive procedure that requires general anesthesia and involves the accompanying surgical complications, and recent studies have identified diagnostic ureteroscopy as an independent risk factor for intravesical recurrence [9–12].

Diagnostic tools, or combinations of tools, that are effective in determining UTUC stage and grade are urgently needed to identify patients for whom conservative management is the most appropriate treatment strategy. Because urothelial carcinoma affects the entire urothelium, the lower and upper urinary tracts share many histological and cytological characteristics in this disease [13]. It is therefore plausible that FISH, which is commonly used in bladder cancer, would be an effective noninvasive, adjunct technique for detecting UTUC. To date, few studies have focused on the utility of FISH for accurately predicting tumor stage and grade in UTUC patients. Johannes *et al*. found that the sensitivity of FISH status of voided urine for predicting low-grade (60%) and high-grade (50%) UTUC was relatively low in a retrospective study with a small patient population [14]. However, Chen *et al*. retrospectively conduced FISH analysis on 94 specimens taken from 43 patients who were monitored for UTUC and found that its sensitivity was higher for predicting high-grade tumors (79%) than for low-grade tumors (41%) [15].

Here, 62.4% of UTUC patients had positive FISH results, which is consistent with previous studies in which the overall sensitivity of voided FISH for detecting or monitoring UTUC varies from 35% to 87.5% [15–19]. Moreover, our data suggest that positive FISH status, which had sensitivities and PPVs of 76.5% and 59.5% for high-grade UTUC and 71.7% and 58.0% for muscle-invasive UTUC, respectively, was the only independent risk factor for advanced UTUC. Due to its relatively high accuracy in predicting these advanced characteristics, voided urine FISH analysis may be helpful in distinguishing tumors best treated using conservative management from those that should be treated with RNU. Radical surgery would be recommended for patients with positive FISH results, whereas conservative surgeries, such as ureteroureterostomy or complete distal ureterectomy and neocystostomy, would be recommended for those with negative FISH results, especially for those with renal insufficiency or a solitary kidney.

The EAU guidelines for UTUC emphasize the importance of tumor multifocality when making decisions regarding the use of RNU or conservative management during treatment. In Chinese populations, the use of traditional Chinese medicine containing aristolochic acid (AA) might affect tumor multifocality, and a previous study of UTUC in Balkan endemic nephropathy patients indicated that malignancy was lower in UTUCs caused by AA [20]. Our previous research also suggests specific treatments that may be most effective for multiple or bilateral UTUC [21, 22], but choosing the best surgical approach remains difficult. By accurately predicting tumor stage and grade, FISH might help improve treatment decisions for these UTUC patients.

Some limitations of our study should be considered when interpreting the results. First, selection bias may have influenced the results of this nonrandomized, retrospective study. In addition, the radical and conservative surgical approaches selected by the surgeons and/or the patients were not based on preoperative FISH results. Finally, we were not able to evaluate the relationship between FISH status and oncologic prognosis due to the short follow-up period. Despite these limitations, this is the largest single-center study to date of the ability of FISH to predict UTUC stage and grade, and is the first report involving Chinese patients. Although a large-volume prospective study should be conducted to confirm these findings, our results suggest that preoperative FISH analysis of voided urine, which accurately predicted UTUC tumor stage and grade, may be a useful tool in guiding treatment decisions for UTUC patients.

## MATERIALS AND METHODS

### Patient selection

A total of 232 patients who underwent surgeries for UTUC in the Urology Department, Peking University First Hospital between January 2012 and May 2016 were enrolled in this study. Twenty-two patients with previous or concomitant bladder tumors were subsequently removed from the analysis. Clinicopathologic data for the remaining 210 patients were retrospectively reviewed. None of the patients had received neoadjuvant chemotherapy. The ethics committee of Peking University First Hospital approved the study.

### Treatment and evaluation

One hundred and eighty-nine patients underwent RNU with an ipsilateral bladder cuff excision; the remaining 21 patients underwent conservative surgery, including ureteroureterostomy and complete distal ureterectomy and neocystostomy.

All pathological specimens were reexamined by a dedicated genitourinary pathologist to confirm the original diagnosis. Tumor stage was assessed according to the Union Internationale Contre le Cancer (UICC) 2009 Tumor Node Metastasis (TNM) classification of malignant tumors. Tumor grade was assigned according to the 2004 World Health Organization classification grading system. Tumor location was classified as ureter, renal pelvis, or both. Tumor multifocality was defined as the simultaneous presence of two or more pathologically confirmed macroscopic tumors in any location. Tumor size was defined as the maximal diameter of the lesion. The status of regional lymph nodes was based on the final pathological specimens. For patients with advanced tumor stages, lymph node metastasis, or postoperative local recurrence, adjuvant radiotherapy or chemotherapy were sometimes administered.

### Criteria for positive FISH

Voided urine specimens from the 210 UTUC patients were analyzed using FISH; labeled probes specific for chromosomes 3, 7, and 17, and the p16 (9p21) genes were used to assess chromosomal abnormalities indicative of malignancy.

**Figure 1:**
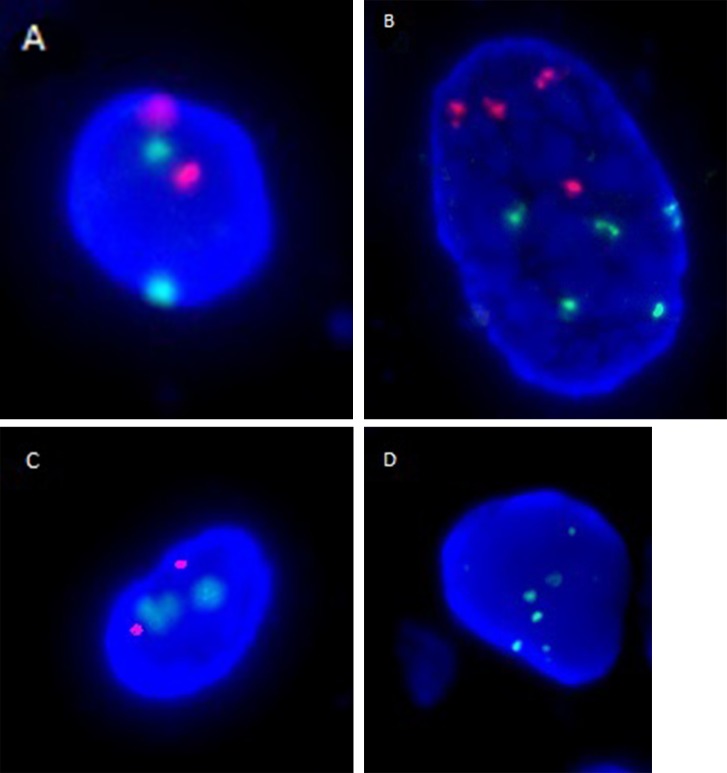
Representative FISH images are shown for (**A**) a normal urothelial cell with diploidy of chromosomes 3 (red signal) and 7 (green signal), (**B**) a cell with polyploidy of chromosomes 3 and 7, (**C**) a normal urothelial cell with diploidy of p16 genes (red signal) and chromosome 17 (green signal), and (**D**) a cell with polyploidy of chromosome 17 and homozygous deletion of p16 genes.

Briefly, FISH signals were examined in > 25 morphologically abnormal, potentially malignant cells per patient. Abnormal signals (Figure 1) were defined as either detection of three or more signals for chromosomes 3, 7, and 17 or the presence of heterozygous (−p16(−1)) or homozygous (−p16(−2)) deletions of p16 gene in the counted cells. A sample was considered FISH-positive for urothelial cancer if at least 4 cells that had two or more abnormal chromosomes (chromosomes 3, 7, 17 or -p16(−1)) were identified and/or if at least 12 cells with -p16(−2) were observed.

### Statistical analysis

Independent-sample Mann-Whitney U and chi-squared tests were used to compare continuous and categorical variables, respectively. Binary logistic regression was used to evaluate preoperative factors. Sensitivity and positive predictive values were calculated using FISH as the diagnostic test and tumor grade (high vs. low) or stage (muscle-invasive vs. non-muscle-invasive) as oncologic outcomes. All statistical analyses were conducted using SPSS version 20.0 (IBM Corp, Armonk, NY). All reported *p*-values are two sided; statistical significance was set at < 0.05.

## References

[1] Munoz JJ, Ellison LM (2000). Upper tract urothelial neoplasms: incidence and survival during the last 2 decades. J Urol.

[2] Roupret M, Babjuk M, Comperat E, Zigeuner R, Sylvester RJ, Burger M, Cowan NC, Bohle A, Van Rhijn BW, Kaasinen E, Palou J, Shariat SF (2015). European Association of Urology Guidelines on Upper Urinary Tract Urothelial Cell Carcinoma: 2015 Update. Eur Urol.

[3] Straub J, Strittmatter F, Karl A, Stief CG, Tritschler S (2013). Ureterorenoscopic biopsy and urinary cytology according to the 2004 WHO classification underestimate tumor grading in upper urinary tract urothelial carcinoma. Urol Oncol.

[4] Messer J, Shariat SF, Brien JC, Herman MP, Ng CK, Scherr DS, Scoll B, Uzzo RG, Wille M, Eggener SE, Steinberg G, Terrell JD, Lucas SM (2011). Urinary cytology has a poor performance for predicting invasive or high-grade upper-tract urothelial carcinoma. BJU Int.

[5] Skacel M, Fahmy M, Brainard JA, Pettay JD, Biscotti CV, Liou LS, Procop GW, Jones JS, Ulchaker J, Zippe CD, Tubbs RR (2003). Multitarget fluorescence *in situ* hybridization assay detects transitional cell carcinoma in the majority of patients with bladder cancer and atypical or negative urine cytology. J Urol.

[6] Bubendorf L, Grilli B, Sauter G, Mihatsch MJ, Gasser TC, Dalquen P (2001). Multiprobe FISH for enhanced detection of bladder cancer in voided urine specimens and bladder washings. Am J Clin Pathol.

[7] Hara I, Hara S, Miyake H, Nomi M, Gotoh A, Kawabata G, Arakawa S, Kamidono S (2001). Usefulness of Ureteropyeloscopy for diagnosis of upper urinary tract tumors. J Endourol.

[8] Keeley FX, Kulp DA, Bibbo M, McCue PA, Bagley DH (1997). Diagnostic accuracy of ureteroscopic biopsy in upper tract transitional cell carcinoma. J Urol.

[9] Lim DJ, Shattuck MC, Cook WA (1993). Pyelovenous lymphatic migration of transitional cell carcinoma following flexible ureterorenoscopy. J Urol.

[10] Luo HL, Kang CH, Chen YT, Chuang YC, Lee WC, Cheng YT, Chiang PH (2013). Diagnostic ureteroscopy independently correlates with intravesical recurrence after nephroureterectomy for upper urinary tract urothelial carcinoma. Ann Surg Oncol.

[11] Sung HH, Jeon HG, Han DH, Jeong BC, Seo SI, Lee HM, Choi HY, Jeon SS (2015). Diagnostic Ureterorenoscopy Is Associated with Increased Intravesical Recurrence following Radical Nephroureterectomy in Upper Tract Urothelial Carcinoma. PLoS One.

[12] Lee JK, Kim KB, Park YH, Oh JJ, Lee S, Jeong CW, Jeong SJ, Hong SK, Byun SS, Lee SE (2016). Correlation Between the Timing of Diagnostic Ureteroscopy and Intravesical Recurrence in Upper Tract Urothelial Cancer. Clinical Genitourinary Cancer.

[13] Melamed MR, Reuter VE (1993). Pathology and staging of urothelial tumors of the kidney and ureter. Urol Clin North Am.

[14] Johannes JR, Nelson E, Bibbo M, Bagley DH (2010). Voided urine fluorescence *in situ* hybridization testing for upper tract urothelial carcinoma surveillance. J Urol.

[15] Chen AA, Grasso M (2008). Is there a role for FISH in the management and surveillance of patients with upper tract transitional-cell carcinoma?. J Endourol.

[16] Chen GL, El-Gabry EA, Bagley DH (2000). Surveillance of upper urinary tract transitional cell carcinoma: the role of ureteroscopy, retrograde pyelography, cytology and urinalysis. J Urol.

[17] Akkad T, Brunner A, Pallwein L, Gozzi C, Bartsch G, Mikuz G, Steiner H, Verdorfer I (2007). Fluorescence *in situ* hybridization for detecting upper urinary tract tumors--a preliminary report. Urology.

[18] Marin-Aguilera M, Mengual L, Ribal MJ, Musquera M, Ars E, Villavicencio H, Algaba F, Alcaraz A (2007). Utility of fluorescence *in situ* hybridization as a non-invasive technique in the diagnosis of upper urinary tract urothelial carcinoma. Eur Urol.

[19] Lodde M, Mian C, Wiener H, Haitel A, Pycha A, Marberger M (2001). Detection of upper urinary tract transitional cell carcinoma with ImmunoCyt: a preliminary report. Urology.

[20] Cukuranovic R, Ignjatovic I, Visnjic M, Velickovic LJ, Petrovic B, Potic M, Stefanovic V (2010). Characteristics of upper urothelial carcinoma in an area of Balkan endemic nephropathy in south Serbia. A fifty-year retrospective study. Tumori.

[21] Fang D, Zhang L, Li X, Xiong G, Chen X, Han W, He Z, Zhou L (2014). Risk factors and treatment outcomes of new contralateral upper urinary urothelial carcinoma after nephroureterectomy: the experiences of a large Chinese center. J Cancer Res Clin Oncol.

[22] Fang D, Liu P, Li X, Xiong G, Zhang L, Singla N, Zhao G, He Q, He Z, Zhou L (2015). Characteristics and treatment outcomes of pan-urothelial cell carcinoma: a descriptive analysis of 45 patients. Sci Rep.

